# Documenting a long-term development model in the slums of Delhi

**DOI:** 10.1186/s12914-016-0088-9

**Published:** 2016-04-28

**Authors:** Martha Morrow, Greg Armstrong, Prarthna Dayal, Michelle Kermode

**Affiliations:** Nossal Institute for Global Health, Melbourne School of Population and Global Health, The University of Melbourne, Victoria, 3010 Australia

## Abstract

**Background:**

Achieving development outcomes requires the inclusion of marginalised populations that have the least opportunity to participate in and benefit from development. Slum dwellers often see little of the ‘urban advantage’, suffering more from infectious diseases, increasing food costs, poor access to education and health care, inadequate water and sanitation, and informal employment. A recent Cochrane Review of the impact of slum upgrading strategies found a dearth of unbiased studies, making it difficult to draw firm conclusions. The Review calls for greater use of process data, and qualitative alongside quantitative methods of evaluation. India is a lower middle income nation with large gender disparities and around 65 million slum inhabitants. The Asha Community Health and Development Society, a non-governmental organisation based in Delhi, has delivered a multi-sectoral program across 71 slums since 1988. This article reports on a mixed-method study to document measureable health and social impacts, along with Asha’s ethos and processes.

**Methods:**

Several observational visits were made to 12 Asha slums where informal discussions were had with staff and residents (*n =* 50). Asha data records were analysed for change over time (and differences with greater Delhi) in selected indicators (maternal-child health, education, child sex ratio) using descriptive statistics. 34 semi-structured individual/small group interviews and 14 focus group discussions were held with staff, residents, volunteers, elected officials, civil servants, bankers, diplomats, school principals, slumlords and loan recipients (*n =* 147).

**Results:**

Key indicators of health and social equity improved over time and compared favourably with those for greater Delhi. The Asha model emphasises rights, responsibilities, equity and non-violence. It employs strategies characterised by long-term involvement, systematic protocols and monitoring, development of civil society (especially women’s and children’s groups) to advocate for rights under the law, and links with foreign volunteers and fund-raisers. Stakeholders agreed that changes in community norms and living conditions were at least partly attributable to the Asha model.

**Conclusions:**

While lacking a control group or complete baseline data, evidence suggested substantial improvements in slum conditions and social equity. The Asha model offers some lessons for slum (and broader) development.

## Background

India’s move in 2008 to lower middle-income status masks the fact that, in 2010, 32.7 % of its population lived on less than US $1.25 per day [1]. The 2013 UNDP Human Development Report [2] ranked India 137th of 186 countries in gender equality. The female labour force participation rate in 2011 was 29 %, versus 81 % among males, and only 27 % of women compared to 50 % of men had at least some secondary education. The distorted sex ratio (a product of illegal sex-selective abortion) is a stark inequality indicator. Among children aged 0–6 years in 2011, there were just 919 girls for every 1000 boys (the natural ratio is approx. 954), a gap that actually expanded since the 2001 Census (927 girls) [3]. Another inequality marker is slum proliferation, with about 65 million living in slums, defined as areas officially denoted as such, or a congested area with an ‘unhygienic environment …, inadequate infrastructure and lacking in proper sanitary and drinking water facilities’ [4].

In 2011 the National Capital Territory (the official name for greater Delhi), with the status of a state, had a population of 16.75 million, of whom an estimated 1.8 million were slum dwellers. A Government of India analysis of 2005–06 data found that 48 % of Delhi slum households had 5+ family members sleeping in the same room (20 % had 7+). As well, < 60 % of dwellings had even one window and 19 % had no access to a toilet facility [5]. Nearly three-quarters used less than the 100 l of water per capita/day deemed a minimum requirement, and 30 % used <50 l [6].

Achieving development targets requires the inclusion of marginalised populations that are more likely to be poor, have difficulty asserting their rights, and have the least opportunity to participate in and benefit from development [7, 8]. Globally, about one-third of urban dwellers live in slums or informal settlements [9]. These populations often see little ‘urban advantage’, suffering disproportionately from infectious diseases, rising food costs, and poor access to education and health care—despite their geographic proximity. Other constraints arise from inadequate water and sanitation, exposure to hazards, threat of eviction, and informal, unprotected employment [10–12]. The 2011 Millennium Development Report noted:

Growing urbanization is outpacing slum improvements, calling for new and realistic national and local targets ([12], p.57).

Little is known about what works best for redressing inequities in urban areas, now home to a growing majority of the world’s people, including more than one billion slum-dwellers. A recently-completed Cochrane Review of the impact of slum upgrading strategies found a dearth of studies made it inappropriate to draw firm conclusions. Among various limitations, the authors noted a lack of detailed documentation of activities and their reach, and of mixed-method evaluations, concluding:

Given the complexity in delivering slum upgrading, evaluations should look to incorporate process and qualitative information alongside quantitative effectiveness data to determine which particular interventions work (or don’t work) and for whom ([13], p.2).

The Asha Community Health and Development Society (Asha) is a non-governmental organisation (NGO) that started in Delhi in 1988. Asha now delivers a multi-sectoral program across 71 slums (approx. 500,000 residents). This article reports on a documentation of Asha’s ethos, processes, and internal monitoring data on health and social equity. The study, conducted between 2010 and 2011, aimed to contribute to the evidence on slum interventions by illuminating plausible links between the organisation’s model and effects at both macro and micro levels.

## Methods

The following methods were used to gather and extract data during and/or after the authors’ two field visits to Delhi:

- Review of program coverage records, printed reports and evaluations, and internal monitoring data on specific health and equity indicators in 23 slum areas
- Informal observation of housing type and size, water systems and availability, drainage, and layout of Asha centres in 12 slums
- Orientation discussions (mostly through interpreters) with Asha staff, organised groups, community members (approx. 50 individuals) (notes hand-written or typed)
- 34 in-depth individual or small group (2–4 people) interviews with 47 people and 14 focus group discussions (FGDs) (4–10 people in each) with 100 people (total 147 individuals). Purposive sample included staff (various levels), women’s and children’s groups, principals, teachers, university students and parents, foreign volunteers/diplomats/supporters, donors, elected officials, slumlords, local authorities, bankers, recipients of land titles and small loans. Semi-structured guides based on themes identified through the orientation visit and secondary literature were used. Most interviews were conducted through an interpreter, with the remainder – and all FGDs—in English or Hindi. Some were audio-recorded and transcribed, but for most interviews transcripts were typed in real time.

Quantitative data were derived mainly from Asha’s routine data collection system, which started in 1994, but covers only permanent residents (not transient populations, whose numbers and residence vary). Data on child, maternal, reproductive and general health, and tuberculosis control were manually entered into registers by staff and volunteers and then transferred to spreadsheets for collation in annual reports. Soft copies became available for specific indicators from 2003 or 2004. For practical reasons we tracked changes from those years to the most recent figures available (2009/10 or 2010/11) (figures were reported annually at mid-year from 2005). For some variables, we compare with available contemporary data for greater Delhi from official sources (including Census and health/household surveys). The quantitative data derived from Asha’s routine monitoring system are not publically available. The quantitative data derived from Asha’s routine monitoring system are not publically available. The Asha director provided detailed annual statistics in the form of Excel spreadsheets to the researchers along with the registers used by staff to enter their routine data. While the raw data are not publically available, summaries of top line findings can be found in Asha’s Annual Reports, which are publically available online.

Qualitative data were collected using question guides that were organised by themes, and were semi-structured to permit clarification, probing and follow-up to explore inconsistencies, seek detail, and test our emerging insights about Asha’s ethos and overall model, and their relationship to outcomes. The following themes guided the qualitative data collection and were developed during an initial orientation visit: organisational structure, evolution and values (ethos); motivations to work/volunteer; community consultation and activities, and perceived change (positive or negative). The transcribed data were deductively and inductively analysed [14] by MM and PD using (as a structure) the themes explored during interviews. The interpretation examined interactions between themes, looking for patterns (and contradictions) within and across sample groups. Asha’s ethos was identified through an analysis of explicit and implicit endorsement of principles and values, which emerged through comments and reported decisions made by individuals and groups. Stories and quotations were used as illustrations.

The study was approved by the University of Melbourne’s Human Research Ethics Committee and the Asha director. Participants were given and were read a Plain Language Statement in English or Hindi, as per their preference. Verbal consent was sought after explaining that participation was voluntary and that comments would not be attributed to a named individual without permission. The same approach was used for children, who at age 15+ are deemed able to give informed consent. An Asha-based community worker was present during interviews.

## Results

### The Asha program

Asha was established in 1988 by Kiran Martin, a paediatrician, following a visit to provide medical care during a cholera outbreak in a Delhi slum. Noting the absence of basic infrastructure (drains, sealed paths, toilets, water, electricity, etc.) mandated under national or local laws and the lack of civic engagement, Dr Martin gathered a team and commenced activities to improve conditions. Today, six programs and a distinctive delivery model characterise Asha’s work in approximately 71 slums across Delhi. This paper focuses on the four programs active at the time of the study:

1. Land rights. Asha-led advocacy persuaded the Government of Delhi to authorise on-site slum renovation with land title in two slums, and relocation with land rights for a community removed during construction for the 2010 Commonwealth Games.
2. Health activities. Asha employs fully trained nurses and part-time doctors to provide primary health care, including maternal and child services, from its community centres and referrals to Asha’s Diagnostic Centre and government hospitals. It trains Community Health Volunteers to offer information and outreach services. Through Asha community groups (women, children, youth) basic health information is shared with peers.
3. Education of children and youth. Asha works with schools, communities, volunteers and donors to boost attendance, supplement learning in English and computers, and support transition to secondary school and beyond.
4. Financial inclusion. Since 2008 Asha has collaborated with nationalised banks in an inaugural scheme of savings accounts and business/education loans.

### Asha’s ethos and model

Direct and indirect evidence was analysed to infer the ethos that guides Asha, and the model (or process) through which it expresses its values. The following key values were endorsed implicitly (and sometimes explicitly):

- universal human rights
- citizenship and responsibilities
- social justice
- non-violence
- accountability

While the founder and director ascribed her principles to Christian values, staff from diverse religious and/or caste backgrounds expressed similar ethics. The equal emphasis on *rights* alongside *responsibilities* (of citizenship) affirmed equality while rejecting paternalism and welfare-dependency, which often characterise NGOs and aid projects—and jeopardise sustainability. Asha’s fundamental aim is the provision—by staff, volunteers and group members—of critical, but minimal, support to mobilise individual and community potential to take effective action based on need. Staff and community respondents agreed that positive, sustainable change must emanate from those who will benefit.

#### Model

The following strategies, discussed in turn below, characterise the distinctive model through which Asha expresses its values:

- Long-term commitment
- Systems, protocols and monitoring
- Strengthening of civil society to address local needs
- Enlisting foreign volunteers and fund-raising.

### Strategy 1: long-term commitment

For over 27 years, Asha’s approach has been to initiate programs in a given *basti* (community or neighbourhood) and to remain there indefinitely. Asha works in slums where the dwellers have squatters rights (i.e. they are permanent residents) and where there are no overlapping services provided by other agencies. Typically, approximately 70–80 % of residents in Asha slums are permanent residents and 20–30 % are transitory residents; Asha primarily focuses on the permanent residents to achieve long-term change. The number of programs and slums expanded as Asha grew in size and capacity. In many (but not all) slums, Asha has operated from the government-funded community centre, offering logistical advantages as well as a symbol of permanence. In other areas, a mobile health van service has been established or building space is rented in the slum. Development organisations often use a short-term approach because funding is rarely given for longer than 3–5 years. By contrast, long-term presence permits Asha greater scope for incremental change and adaptation based on observed outcomes, according to staff.

In the early days, Asha staff were met with community disinterest, wariness, even hostility—often lasting months—towards outsiders coming ‘to help’. Several attributed this to a protective instinct among an exploited population subsisting on the margins, familiar with the often empty promises of politicians visiting only at election time. Asha’s first efforts were described as a balance between establishing human relationships and offering tangible responses, such as child immunisations. They invited women to chat in laneways about their lives and needs and—if interest seemed sufficient—to attend group meetings that would lead to joint lobbying efforts to obtain their rights (among other things). Most women (and their husbands and in-laws) were bemused by the very notion of organised meetings (an activity most had never experienced), seeing it as a ‘waste of time’. The staff continued to appear daily, offering practical advice and health care. Finally, a few women overcame their hesitancy and joined groups that began to learn how to lobby for infrastructure improvements. The next challenge for staff was attendance drop-off when early lobbying attempts did not yield the swift results expected by the community.

If the women stopped going to [make requests to the government] … Asha would continue to do so, and eventually the community returned to join in. (Asha senior staff member)

Interviews with numerous residents and staff confirmed Asha’s constancy, personal presence and visible actions, which gradually generated the trust essential for success. Perhaps the best evidence of Asha’s long-term commitment is the acceptance of hundreds of Asha slum students into tertiary education since 2008 (see pp 20–21), a culmination of collaborative efforts between Asha and the community that literally span the lifetime of these students.

### Strategy 2: systems, protocols and monitoring

The value of accountability is expressed through its guidelines, systems of record keeping, and regular progress reviews. From the early days, Asha has gathered data to enable it to respond to—and measure—change. Today its data gathering and management system is sophisticated, covering both process (content and reach of activities) and impact (change in behaviour, health status and specific measures of equity). Data are gathered regularly at basti level and posted on Asha centre walls, allowing scrutiny by the community, including by women’s groups while determining priority needs. Asha headquarters collate data across centres, enabling it to identify changes in health-seeking that may indicate disease outbreaks, and tracking of process and outcome indicators. Data are used for decision-making and for reporting to government, donors and the community. More informally, Asha uses staff exchange visits to other slums as a type of evaluation, and also to generate new ideas and reflection on weaknesses and strengths.

### Strategy 3: strengthening civil society to respond to local needs

Building civil society relies upon the emergence of grassroots leaders able to galvanise community support. This was a challenge in Delhi slums, where women tended to be illiterate or poorly educated and leading restricted lives, often leaving home only for marketing (if at all). The 2005–6 National Family Health Survey found just 37 % of married women participated in decision-making on health care, large or daily household purchases, and visiting their relatives; 20.5 % did not participate in any. Just 51 % of married and unmarried were ‘permitted’ to go alone to the market, 48 % to a health facility, and 38 % to travel outside their community. Lowest rates were among the less educated and poorest [15].

‘Community participation’ and ‘strengthening civil society’ are rarely defined clichés in development circles. Asha’s most visible feature is the breadth and dynamism of its organised groups; these are the lynchpins of citizenship, generating community action and control by those typically lacking autonomy. Asha’s most active associations are those for women (*Mahila Mandal*) and children aged 6–14 years (*Bal Mandal)*, although it also has teenage peer education groups

#### Community groups: structure, operations and official registration

One of Asha’s first steps in a new basti is to identify women with leadership potential to form a Mahila Mandal. After it is well established, Bal Mandals are similarly created. Each Mandal has approximately 35 members, with the number of groups per slum depending on population size. Group structure includes:

- Weekly meetings
- Payment of dues (Rs 10 per month [USD 0.16 at April 2015])
- Discussion of community needs and responses
- Building of skills and knowledge by Asha staff
- Social gatherings
- Reporting on action taken.

A rigorous selection process is employed to confirm membership. Aspirants must: attend meetings and pay dues regularly for up to three months; accept regulations and shared decisions; and donate time, energy and talents. These requirements ensure members take membership seriously, learn the process of compromise, and attain (literal) ownership through committing scarce financial resources. Asha uses detailed protocols to train groups about human rights and equity, basic health care, gender discrimination, non-violent action and effective advocacy, and accountability, rules, and shared responsibility for action. Groups function without financial inputs from Asha, and members learn the advantages and satisfaction of making common cause.

Perhaps most singular is the Lane Volunteer system, whereby members visit approximately 25 households regularly to identify needs and convey important information, such as rights under law or disease outbreaks. Acute overcrowding and insufficiency of space in slums often generates competition between neighbours that is exacerbated by the artificiality of ‘communities’ of migrants from several states, sometimes with no common language and varied religions and castes. Familiarity with 25 households fosters recognition of shared interests and diminishes tensions that may obstruct unified action, as expressed by two Mahila Mandal members:

I can feel their feelings, which can help me to reach them better.

Earlier we weren’t able to approach the authorities because we were all separated, but now we are united, and we can.

Bal Mandal members reflected on similar changes.

What is special [in] Bal Mandal is that whatever we do, we do it all together.

Earlier we were only friends with children in our lanes. Now we are friends with the entire basti.

The system provides an identified ‘go-to’ person for individuals or families in crisis, who otherwise may be left to their own devices. It also aids Asha in monitoring of changing needs in different slums, organisational planning and higher-level advocacy.

Asha enhanced the sustainability of community groups in 2002 by obtaining registration of Mahila Mandals under the Indian Societies Registration Act, which requires democratic elections, financial auditing, and regular meetings. Registration offers autonomy and public status, and Mahila Mandals are often seen as useful partners by local government (possibly explaining why several authorities we interviewed had mobile phone numbers of group leaders). Official status connotes self-reliance and the expectation of future performance on behalf of the community.

#### Learning about rights, responsibility, non-violence and effective advocacy

Asha aims to transfer not only skills but its ethos. Group members come to understand rights under Indian and international law, including rights to education and the illegality of child labour and child marriage, which persist widely due to custom and poverty. Children are taught about hygiene, dental health, infant nutrition and growth monitoring, management of the main causes of child mortality and the consequences of tobacco and alcohol misuse. They also learn ways to communicate messages. Stress, time management and exam preparation are covered to strengthen their capacity for formal education.

Mahila Mandal curriculum is broader, covering additional health topics and social issues in detail. Emphasis is placed on the social basis of gender roles, gender equity and the distorted sex ratio, discrimination against the girl child, sexual harassment/assault and domestic violence. Both groups learn about legal protection, as well as Delhi Government schemes for the girl child, widows and the elderly, and obligations of governments to provide basic services. Respondents recalled having learned—and practised—pragmatic tips for advocacy success, such as maintaining eye contact and erect bearing to convey self-confidence, and the need for persistence.

They don’t listen if we just go once. We went repeatedly, over two or three months. (Mahila Mandal member)

Asha also emphasises what it calls ‘active peace-making’ as intrinsic to effective, sustainable social change. Members are enjoined to treat each person in this hierarchical society with equal respect, and to make allowances for the influence of upbringing on world views and values. Asha aspires to fellowship, even intimacy, between castes and religions in a nation with sectarian allegiances and communal violence. Staff lead by example, publicly sharing inter-caste meals, and demonstrating affirmation to all. Observation of numerous groups and Asha centres showed women and children from Muslim and Hindu backgrounds (including different castes) working, playing and eating together in apparent harmony. Community members, local officials and police chiefs agreed there had been no communal strife in Asha slums, despite occurrences in Delhi and nationally.

When we first started … segregation existed … Going to lobby together as a group can be a powerful experience… [and] an act as small as eating something together … breaks down … barriers. (Kiran Martin, Asha founder)

Groups learn that seeking rights need not involve vocal taunts, hectoring and even violence, which often characterise mass action. Asha identifies ethical and strategic reasons to act with grace towards those with decision-making power. Members are encouraged to invite slumlords, police chiefs, Councillors, etc. to family or community events, and groups invariably write thank-yous and invite donors and officials to celebrate achievements. These quotes from two respondents reflect the contrast perceived between Asha and other NGOs:

There is some difference with Asha … [Asha] approaches me more personally than the other ones. The relationship is direct. (Civil servant)

Asha [is] doing much more than I do… In Asha, people work like family members … The [purpose of other NGOs] is the same, but they don’t feel others are like their brother, sister or friend. (Parliamentarian)

Slumlords typically exploit residents and block actions (like those performed by Asha groups) that could reduce their power. Yet some have warmed to Asha.

Whatever they have done has benefited us … Only Asha has worked with the poor like this. (Slumlord in an Asha community)

#### Advocacy efforts and results

Asha does not raise money to fund services and infrastructure mandated under law. Instead, the Mandals elicit community needs and then direct their efforts at government departments, typically through multiple letters and mass visits. In 2009–10, 63 women’s groups collectively made 444 lobbying visits; 54 children’s groups made 177 visits. Asha groups have successfully lobbied to ensure residents were issued with Ration Cards for access to basic commodities. These cards can also be used as proof of residency for school enrolment, bank loans, etc. In one slum, the group agitated to increase ‘ration shops’, making goods more available. Lane Volunteers encourage birth registration, partly to guarantee the child’s eligibility for normal entitlements, especially for girls, who receive Rs 100,000 on completion of year 12 if births are registered within two months.

One manager of a toilet complex was forced to stop charging women to use it following Mahila Mandal intervention. Elsewhere, a group physically obstructed work until contractors met quality standards for lane paving. Slums often lie along railway lines used as de facto toilets at great risk to residents. One group lobbied the local elected official for years until he visited and then authorised construction of a toilet block. And groups in several slums convinced Delhi Police to establish posts within or near slum boundaries, reducing violent episodes and forcing out illegal alcohol shops. Progress has occurred in many slums in relation to water, sewerage and paving. Mahila Mandals also fill gaps where governments do not respond, such as accompanying new residents for school enrolment and spouse mediation.

People think of the Mahila Mandal as a forum … if they are having problems. They come to us to get their issues resolved. (Mahila Mandal member)

Such activities are supported by dues and donated time, but some Mahila Mandals secured donations from elected officials and shop-keepers by explaining their needs

FGDs with Bal Mandals revealed numerous examples of impacts from raising awareness, assisting people to receive entitlements, and lobbying authorities. Groups often hold poster competitions and street theatre about health and social inclusion, highlighting dangers of alcohol and tobacco, child marriage and domestic violence. Groups also decide on spending dues, e.g., ‘From the money we collect we buy eggs, milk and bread for the poor people in our basti’. One member recounted successfully persuading one mother to send her young boy to school rather than keep him working in the family’s food stall.

Sometimes parents don’t know that until the fifth grade … pupils get free tuition, free clothes and books and mid-day meals. We go and explain this to parents who aren’t sending their children to school. (Bal Mandal member)

One group helped widows obtain identification cards needed to start collecting their pensions for the first time, and another described what happened when rubbish started to be dumped outside the Asha centre where children studied.

So we wrote an application and went to the authorities requesting them to do something because the smell was so bad we couldn’t study. Now the garbage is regularly removed. (Bal Mandal member)

Asha reports showed that all women members and many young people had personal bank accounts, and some had loans. Even illiterate women began to assume leadership roles within families and laneways as they transferred useful knowledge and supported slum improvements. Efforts do not always bear fruit, but members learn to work collaboratively, persevere, and learn new ways to seek redress.

We put in several applications to close the liquor store near our basti. It has now been closed but alcohol is still being sold illegally. So, we’ve put in another application to address this problem. (Bal Mandal member)

### Strategy 4: enlisting foreign volunteers and fund-raising

One area that partially diverges from Asha’s emphasis on pressuring governments to fulfil their obligations is in subsidising of some health services in Asha clinics. Asha also needs to generate income to cover salaries of senior staff and, since 2008, to support students undertaking tertiary education. The latter is critical given parental reluctance to postpone a child’s entry into the workforce to supplement household income. Asha has established supporter groups in various countries which do fund-raising, often combined with activities to expose western communities to slum conditions and needs. As well, Asha receives numerous short- and longer-term volunteers who teach English and basic computer skills (both are critical for educational advancement), or offer specialist medical services or staff training. In interviews, volunteers and regular visitors acknowledged their evolving awareness of differences between the ‘charity model’ and the Asha model, under which communities take primary responsibility for social change. A British ‘Friend of Asha’ had brought her family repeatedly to see change in a particular slum.

They had no toilet facilities [and] had to go to the toilet in a park. A little girl had been murdered when she went to the toilet in the middle of the night, and the people had great difficulty in getting that investigated. So the women’s group gathered and just sat in the road. They stopped the traffic so the police would take action… People ask me, how do you feel seeing all that poverty? I say, what I see is the difference Asha is making, lifting them out of poverty. It excites me hugely. (British fund-raiser for Asha)

While the funds and volunteers have provided a measure of stability for Asha, reliance on such sources creates vulnerability to national and global events.

### Health and equity impact data

This section presents our analysis of data on maternal-child health and proxy measures of social equity and rights within India’s sociocultural and economic context.

### Maternal and child health

Analysis suggests that the health of mothers, babies and children in Asha slums was substantially better than for the municipality of Delhi, which includes large middle class populations. During 2009–10, nearly 100 % of pregnant women in Asha slums had ≥3 antenatal checks, gave birth in hospital or with a trained midwife, and breastfed immediately after birth. In Delhi in 2007–08, only 72 % of pregnant women had ≥3 checks, 72 % delivered with skilled assistance, and 29 % breastfed immediately after birth [16]. Less than 10 % of newborns in Asha slums had low birth weight (<2.5 kg) versus 26.5 % across Delhi in 2005–06 [17].

**Fig. 1:**
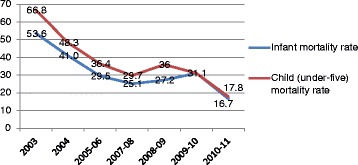
Infant and child mortality rates over time in Asha slums (deaths per 1000 live births)

There was a marked decline in both infant and child mortality in Asha slums (see Fig. 1). Infant mortality dropped from 54^1^ per 1000 live births in 2003 to 31 in 2009–10, and more recent figures (2010–11) suggested a decline to 17^2^. By comparison, infant mortality in Delhi fluctuated: 32 in 2000, 28 in 2005, 36 in 2007, and 33 in 2009 [18]. Child mortality in Asha slums declined from 67^3^ per 1000 live births in 2003 to 18^4^ in 2010–11; Delhi’s most recent comparable estimate was 37 in 2009 [18].

Nearly 100 % of infants in Asha slums were recorded as fully immunised, and 95 % had two doses of vitamin A supplementation, versus 55 % given at least one dose in Delhi [16]. There is a lack of baseline comprehensive data on child malnutrition across Asha slums in general. However, Asha introduced its program in Zakhira slum in 2004, and has continuous monitoring data in softcopy format from then to the present day. At baseline, 15 % of children <5 years were severely malnourished and 61 % were moderately malnourished. By 2009–10, just 3 % were in either category.

### Education

Educational achievement, a critical contributor to life chances, was substantially greater in Asha slums, where 90–95 % of children aged 5–10 were attending primary school and 60–70 % secondary school at the time of the study. The most comparable data for Delhi (2005–06) showed 23 % of females and 11 % of boys aged ≥6 years had had no education [19]. Among secondary students in Asha slums, 15–20 % had reached class 12 and 38 % of this sub-group was accepted into tertiary study, many at prestigious Delhi University. The number of students from Asha slums that were accepted into tertiary study each year has been rising consistently; 58 students were accepted in 2008, 157 in 2009, 173 in 2010 and 190 in 2011.

### Child sex ratio

By tradition, parents of girls must feed and clothe them, provide dowry and pay for their wedding, after which the bride moves permanently into the husband’s home. These customary costs and disadvantages help explain sex-selective abortion and neglect of female children. Delhi’s child sex ratio (0–6 years) at the 2011 Census was 866 girls per 1000 boys, virtually unchanged since 2001, but even more distorted than in 1991 (915). Delhi has the fourth worst sex ratio among all states [20]^5^. By contrast, in 2010–11 in Asha slums there were 956 girls to 1000 boys among children 0–5 years, which is the natural ratio. Although baselines for slums do not exist, Asha’s figure indicates striking shifts in sociocultural norms in the context of acute poverty.

### Perceived impacts and relevance of the Asha model

In this section we report qualitative data about the personal impact of participating in Asha groups, and consider some broader lessons for slum development.

During interviews and FGDs almost every Mahila and Bal Mandal member described changes they had observed in themselves which led to their adopting new roles inside and outside the home. Women tended to focus on growth in self-confidence, autonomy, participation and expanded horizons, as illustrated by the following:

I have become much stronger … Asha has changed me to allow me to work on my own, with [Asha] staff in the background only.

Because we used to stay at home, we didn’t have much knowledge about anything. We didn’t even value education.

Earlier we needed a man for everything. Now we can do a lot … on our own.

And even though we are illiterate, we know how to go to the bank to deposit money.

After Asha came we interacted with different people. We learned so much. Before, I didn’t go out. I couldn’t speak to anyone, even my husband or father-in-law. But now I’m a tigress! I used to cover my face, but not anymore.

We were very shy. Now we have a voice.

Whereas before I was confined to myself, eating and sleeping, now I have my eyes open and I can see and can help other people. Helping others, I like it.

These observations by Bal Mandal members reveals that even quite young people recognised personal growth, positive expectations and strength in unity.

Earlier we used to never feel like studying. We hardly attended school. We would just go up to the building, but then run away.

When people… talk to my mother about my work, she feels very proud.

At first the community took us lightly when we gave them information about TB, but then they began to take us more seriously. I had problems with some people who drink, who would abuse us, but now they have accepted us.

Through the exposure children get at a young age to meet the [parliamentarian], police chief, and all, the child feels, ‘I am in such an important place’. Children learn about the roles these people have. And as part of Bal Mandal, we can also address the problems in our own community. We know where to go for help. If an elderly person isn’t getting a pension, we know who should be responding. If I wasn’t a member or there was no Bal Mandal, I would only know a few people in my lane… We wouldn’t have been able to get people to sign up for a signature campaign.

Asha’s model of supporting communities as citizens to battle for rights, rather than doing it for them or offering handouts, offers the potential for sustainable change that was recognised by several respondents, including this male resident of an Asha slum which received land titles.

If anyone wants to help people …, instead of giving small gifts that last a short time, give these lasting things. This land is more precious than gold to us. If we had been given any other thing it would be gone by now, but this will last for generation after generation… If the Australian government wants to help the poor they should do it this way…[Whatever] we’ve learned from the training will go on and on. (Community member)

Other evidence of sustainable change is reflected in the child sex ratio and the equal numbers of girls and boys from Asha slums in tertiary education. This shift in gender attitudes was noted by a local (male) elected official.

Asha’s most important work is education, health care, medicine, and the empowerment of women. Women are now much more aware. In olden times, the men didn’t like it, but now it’s okay; women have the right to come forward and the mindset has changed. Okay, one hundred percent of men should think this way, but not all of them do. About half of them do. (Councillor)

Two senior western diplomats who knew Asha well were asked to comment on the organisation’s model, and its relevance for development. Both saw merit in terms of effectiveness and sustainability, and felt it offered lessons for practitioners and donors. One of them, who focused particularly on Asha’s emphasis on rights with responsibilities, put it this way:

The emphasis on empowerment and looking to women to play a leading role was impressive. Also I was impressed that it’s not something for nothing; there is a contribution from the recipient. It makes a difference to the mindset. Also the emphasis on education, because it is the most effective accelerator of upward social mobility and Asha has recognised that. Taken together, it was based on solid first principles, and works in action… The thing that really struck me was their level of self-confidence about their own lives that would never have happened without Asha, and a sense of social responsibility. It isn’t just what I can get out of it, but what I can give too. In terms of Asha’s most important contribution, you can see it in the lives of people it’s affected, but [also] … the template it establishes for dealing with challenges outside of India. I think wherever you see something that works, it makes sense to see how this can be applied elsewhere, especially at a time when the whole project of development assistance has had a patchy outcome. (Senior diplomat)

## Discussion

Urbanisation and proliferation of slums present deep challenges for equity, health, environment and social cohesion. Governments, agencies and communities are struggling to find affordable and effective ways to address these threats. The Cochrane Review of slum upgrading programs found strong evidence of impact only in relation to diarrhoeal diseases and water expenditure. Others were difficult to attribute and the authors propose process evaluations and qualitative methods alongside quantitative studies to better illuminate local context and perspectives [13].

This study utilised quantitative methods to document a range of outcomes in slums where Asha has worked for up to 27 years. Through analysis of indicators considered most relevant for health and equity, Asha data consistently outperformed comparable figures for greater Delhi, which includes a large middle class population. The positive relationship between these indicators, such as skilled attendance at birth and neonatal survival [21], and birth weight and child nutritional status [22], provides a level of internal corroboration, Qualitative methods illuminated widespread perceptions of personal growth, community cohesion and improved living conditions, providing further corroboration of quantitative results. The natural sex ratio reported in Asha slums may be the most powerful proxy for social change, as it suggests an optimism that girls will participate actively in economic and social spheres. A similar observation can be made about parental approval of daughters’ higher education, traditionally rejected due to norms enjoining women to remain within the home, with limited opportunities for outside interaction.

Asha records and our observations present clear evidence of improvements that create more favourable conditions for health, employment, and poverty reduction. Construction of toilet blocks (with priority access for girls and women) removes the necessity to use risky sites like railway lines or distant, unlighted spaces vulnerable to physical and sexual attacks. Some slums have piped water, and others more regular deliveries by water trucks. Paved laneways and improved sewerage facilitates easier transit to schools, markets and jobs, and reduced transmission of fly- and water-borne disease. Increased police presence within slums helps ensure security and order. A wide range of informants agreed that these initiatives largely occurred through women’s and children’s groups trained in advocacy by Asha.

The establishment of Asha centres at community level offers convenient (and more affordable) health care, health education, tutoring and classes in computers and English. Uptake of savings accounts and greater availability of loans offer security (and incentives) for savings, and the possibility of small business development. While land title rights and house renovation occurred in just two Asha slums, they now approach middle class in employment, housing, and visible affluence. Finally, the enrolment of nearly 600 Asha slum students during 2008–11 in tertiary institutions—including Delhi’s most selective—represents years of inputs: tutoring, mentoring, practice exams and conversations with students and parents. Observation of cramped housing with intermittent electricity and constant noise in slums reveals the magnitude of this achievement.

Because Asha program components were introduced incrementally over many years, and not every activity occurred in every slum, it is not possible to control for single versus synergistic effects across the slums as a whole.

The Cochrane Review of qualitative data from included studies identified a preference for synergistic components to be implemented simultaneously ([13], p. 48), which may imply an assumption that this would amplify positive impacts. Our qualitative analysis suggested that change at both individual and community levels was mutually reinforcing, enabling the growth and consolidation of civil society. Respondents perceived increased cohesion and reliance on advocacy through the instruments of civil society, and away from strife arising through competition for space or water, or religious/linguistic/caste distinctions). Children spoke in similar terms about positive peer relationships and joint advocacy through their Asha involvement.

Perceived change was greatest among female slum residents, whose new, and enduring, relationships and public roles contrast with lives spent within the four walls. Across north India *purdah* (literally, veil or curtain) is practised by both Hindus and Muslims to safeguard family ‘honour’ by ensuring and demonstrating female sexual purity. For married women, purdah typically involves covering the face and head with the sari cloth in the presence of men (in public) and in-laws (at home), and even staying behind a wall or door in the presence of in-laws. Where movement outside home is unavoidable, women should show signs of purpose (such as carrying shopping bags) and symbols of marital status in their dress [23, 24]. Phadke [24] and Still [25] note that calls for greater orthodoxy and focus on female ‘honour’ among both major faiths have emerged over the past two decades, reinforcing constraints on movement at the very time female education and employment opportunities have increased. Some families withdraw daughters from school at puberty to pre-empt potential loss of reputation; among youth aged 15–24 in 2005, just 30 % of females had completed lower secondary education, versus 57 % of boys in Rajasthan, north India [26]. Yet girls and young women in Asha slums walk to school or get buses to university, and many participate in entrepreneurial and community life. A mixed-method study in Chandigarh, India, found that property rights (as provided in two Asha slums):

increase women’s participation in decision making, access to knowledge and information about public matters, sense of security, self-esteem, and the respect that they receive from their spouses [27].

The gradual dissemination within women’s and children’s groups of information on health, human rights, the process of civil and non-violent advocacy, the need for persistence, and the responsibility of members towards the wider community seemed to cement bonds of shared purpose and mutual support that were essential to successful advocacy. Respondents attributed their achievements to:

- Asha’s presence over the long term, offering guidance that gradually diminished
- Staff sensitivity to the need for self-respect by offering opportunities for learning and decision-making
- Mechanisms (based on Asha’s ethos) to build residents’ personal capacity and confidence, along with awareness of the value of joint advocacy.

The 2011 Asia-Pacific report on health inequity notes that ‘awareness and advocacy from active civil society’ is among five approaches associated with slum reduction and upgrading ([28], p. 42). The hundreds we interviewed saw themselves as largely responsible for improvements in infrastructure and community cohesion, even if Asha had mentored them. They share greater optimism as an increasing number secure small loans, and youth enter tertiary education. With these conditions and life skills, residents can continue to seek their rights, even without Asha.

## Conclusions

The complexity of discerning change and its attribution through slum upgrading remains a challenge in dense urban settlements with transient populations often lacking identity documentation or ownership of dwellings. Challenges are particularly daunting for evaluating long-term development programs that expand their coverage and alter interventions over time, as in the case of Asha. Understanding, documenting and accounting for the synergies that arise with increased range of activities is inherently difficult [13]; so too with external confounders, such as positive changes in government policy or the role of a powerful stakeholder that may uphold, or undermine, the interests of slum residents. Among 10,488 studies identified for the Cochrane Review, just five were selected for main analysis, yielding mainly equivocal results. Our study would not have met the selection criteria because it was neither a randomised-control trial, a controlled before and after study, or an interrupted time series design. It was also limited in reliance upon internal data that could not be objectively verified, and possible social desirability bias from respondents.

Although these limitations are acknowledged, our design offered process and qualitative data in tandem with analysis of impact data, which the Review authors see as useful for assessing dynamic programs in complex settings. We also argue that less tangible features, such as organisational philosophy or ethos, and the community’s own perceptions of meaningful change, are relevant for slum upgrading program evaluation.

### Availability of data and materials

The quantitative data derived from Asha’s routine monitoring system are not publically available, other than in summary form in Annual Reports on the Asha website. The data were provided to researchers by the Asha director in the form of Excel spreadsheets and we were also provided with the registers used by staff to enter this routine data. Anyone wishing to access this data should contact the Asha director.

## Abbreviations

Asha: asha community health and development society
FGD: focus group discussion
NGO: non-governmental organisation

## Footnotes

1 Based on 1286 live births and 69 deaths of children aged under 1 year.

2 Based on 898 live births and 15 deaths of children aged under 1 year.

3 Based on 1286 live births and 86 deaths of children aged under 5 years.

4 Based on 898 live births and 16 deaths of children aged under 5 years.

5 Census of India 2011. Office of the Registrar General and Census Commissioner, India.
